# Transportation Assessment in Simulated Curved Canals after preparation with Twisted File Adaptive and BT-Race instruments

**DOI:** 10.4317/jced.54220

**Published:** 2017-09-01

**Authors:** Carlos-Vieira Andrade-Junior, Nilton-Dessaune Neto, Renata-Costa-Val Rodrigues, Henrique-dos Santos Antunes, Mariana-Teixeira-Maneschy Porpino, Júlio-Cesar A. Carvalhal, Luciana Armada

**Affiliations:** 1 PhD, Department of Endodontics, Faculty of Dentistry, Estácio de Sá University, Rio de Janeiro, RJ, Brazil; 2 PhD, Department of Health, Dentistry Division, Southwest State University of Bahia, Jequié,BA, Brazil

## Abstract

**Background:**

This study compared the incidence of deviation along curved canals after preparation with two nickel-titanium (NiTi) rotary systems, Twisted File Adaptive and BT-RaCe.

**Material and Methods:**

Forty resin training blocks with curved canals were filled with ink and divided into two groups according to the instrumentation technique. Preinstrumentation images were acquired by using a stereomicroscope. The canals were up to an instrument #35/0.04. Postinstrumentation images were captured using the same conditions, and the images were superimposed. The amount of resin removed was measured at 8 different points, beginning at the apical terminus of the canal. Differences in the mesial and distal aspects were measured to evaluate the occurrence of deviation. The Student’s-t test was used for comparison of the intragroup deviation. Intergroup analysis was performed by using one-way ANOVA for each level. For multiple comparisons, the Bonferroni test was used and a cutoff for significance was set at 5%.

**Results:**

Intragroup analysis showed that both instrumentation techniques promoted some deviation at all levels. BT-RaCe showed significantly lower deviation at 0 and 4-mm levels than Group Twisted File Adaptive (*p*<0.05). On the other hand BT-RaCe showed worse performance at level 6.

**Conclusions:**

The results of this study demonstrated that none of the NiTi tested systems was able to instrument curved canals simulated in resin blocks without some deviation during the preparation. There is still need for improvement in the instruments manufacturing aiming the better performance of endodontic files in curved root canals.

** Key words:**BT-RaCe, Curved root canals, Deviation, Twisted File Adaptive.

## Introduction

Chemomechanical preparation is intended to promote root canal cleaning, disinfection and shaping. Root canal preparation involves enlargement and shaping of the root canal system, preserving the location of the apical terminus, however, in curved canals, it may not be an easy task to attain an adequate shape (1,2). Technological advancements in nickel-titanium (NiTi) instruments have led to new concepts of root canal shaping (3-6).

The iatrogenic transportation of curved canals could compromise the prognosis of the endodontic treatment in infected cases since some areas could not be properly prepared and disinfected. Therefore, to maintain the root canal preparation as centralized as possible, the use of NiTi instruments is strongly recommended (7). To maintain the root canal preparation as centralized as possible, the mechanical property - flexibility - of NiTi systems, enables a curved root canal preparation that is wider in the apical portion, more centered and with fewer deviations (8).

Recently, several new endodontic NiTi systems have been developed with different characteristics, such as: cross-sectional shape; taper; number and helical angle; kinematics; in addition to changes in metallurgy properties to enhance the mechanical properties of NiTi instruments (7,9,10). Two NiTi systems have been highlighted in the market. One is Twisted File Adaptive (TFA), developed by SybronEndo (Orange, CA, USA), wich is proposed for use in a combined continuous rotation and reciprocating motion, with three unique design features, namely R phase heat treatment, twisting of the metal and special surface condition. This adaptive technology and the twisted file design is claimed to reduce the risk of instrument failure, increase flexibility and canal centering ability (11-13). The other system is BT-RaCe (FKG Dentaire, La Chaux-de-Fonds, Switzerland), which was recently introduced. According to the manufacturer, it presents a “Booster Tip” (BT-Tip) for improved efficacy (14). These two systems were selected for this study because they are recently developed and according to the manufacturer, both systems have improved flexibility, showing less transportation during the chemomechanical preparation.

Until now, no study has analyzed the performance of these two endodontic rotary systems during the preparation of curved root canals. Therefore, the aim of this study was to compare the incidence of deviation along simulated curved canals in resin blocks, after instrumentation with TFA and BT-RaCe NiTi rotary systems. The null hypothesis was that there would be no difference in the incidence of deviation in curved canals after instrumentation with the two NiTi rotary systems tested.

## Material and Methods

For this study, 40 simulated curved root canals in clear resin blocks with a 2% taper, 10-mm radius of curvature, 70º angle of curvature, and 17-mm length (Dentsply Maillefer, Baillagues, Switzerland) ISO #15, 0.02 tapered, were used and randomly divided into two groups with 20 blocks each. All the simulated canals were filled with ink by using an insulin syringe. Before any instrumentation procedure, a round silicon base with a rectangular slot fitting the microscope base was positioned under a color stereomicroscope (1005t Opticam stereomicroscope; Opticam, São Paulo, Brazi). A digital image was captured of each specimen before instrumentation by using the software Leica Application Suite 3.6 (Leica), at 1.25× magnification, and saved in a TIFF format file (15). After the instrumentation procedures, all blocks were imaged again following the same protocol. The working length (WL) was established at the terminus of the artificial canal (0 limit) and all instruments were used up to this limit. The canals were initially irrigated with 2 mL tap water to remove the excess dye. An endodontic specialist performed the negotiation and glide path of all the canals with stainless steel K-file (Dentsply Maillefer, Baillagues, Switzerland) sizes 10 and 15, all being used in circumferential filing motions. In both systems 3 instruments were used; each file was used with four pecking motions in an amplitude of 3 mm. Apical patency was confirmed with a hand stainless-steel K-file size 10 between each rotary instrument size. Preparation with all systems was completed with file tip #35 and taper 0.04mm/mm. For irrigation, a 5 mL BD Plastic syringe was used with a NaviTip (Ultradent Products, South Jordan, USA) of 30G and length of 21 mm, from a distance of 3mm short of the WL. No lubricant was used during preparation. Irrigation was performed with 2 mL tap water, after each instrument size and 1 mL after each patency instrument, totaling 15 mL per canal for all groups.

-Preparation Techniques per Group

The TFA group

**Figure 1:**
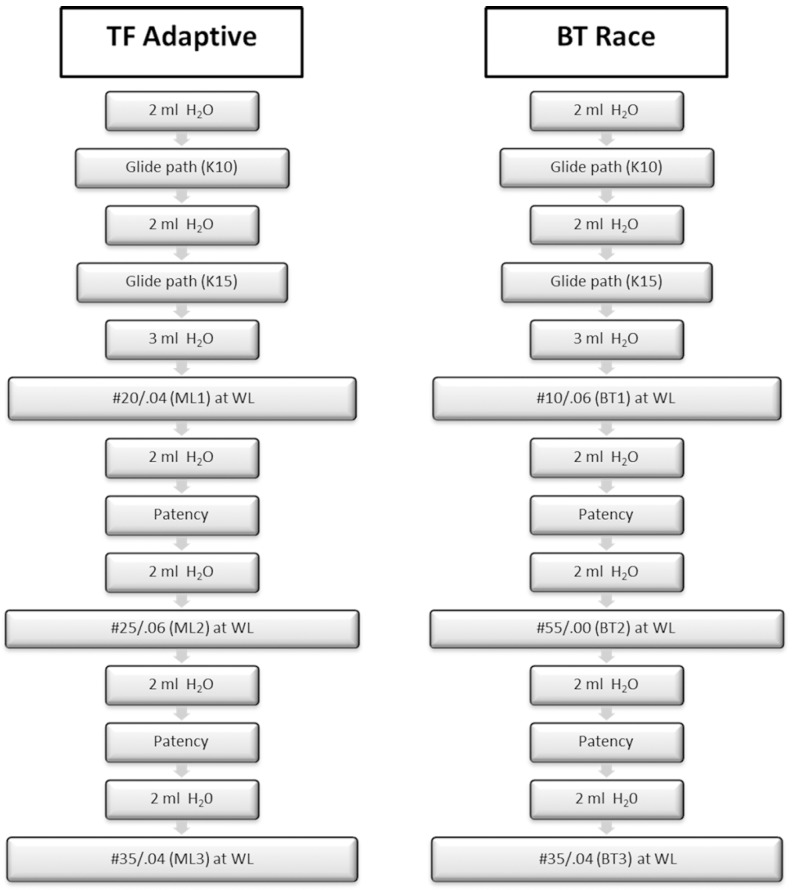
Flowchart of the instrumentation protocol during preparation of simulated curved canals. TFA:Twisted File Adaptive, WL: Working Length.

In this group, the canals were prepared with the TFA system with instruments SM1, SM2 and SM3, files #20/.04, 25/.06 and 35/.04 respectively. The TFA instruments were coupled to the Elements Motor (Axis, SybronEndo, Texas, USA) at the TFA setting suggested by the manufacturer. Flow chart of the canal preparation with TFA is illustrated in Figure 1.

The BT-RaCe Group

In this second group, the canals were prepared with the BT-RaCe system with files BT1, BT2 and BT3 #10/.06, 35/.00 and 35/.04 respectively. The BT-RaCe instruments were coupled to an endodontic motor (VDW, Munich, Germany) at the setting suggested by the manufacturer. Flow chart of the canal preparation with BT-RaCe is illustrated in Figure 1.

Image analysis

**Figure 2:**
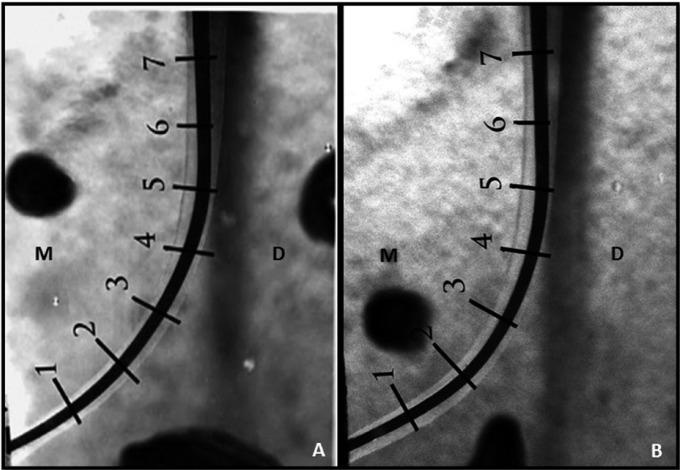
Superimposed preinstrumentaion and postinstrumentation images of representative specimens of the groups TFA (A) and BT-RaCe (B). TFA:Twisted File Adaptive, M: mesial, D: distal.

After instrumentation, all specimens were repositioned in the apparatus and photographed as described above. Photoshop software (CS5 Extended, version 12.0.4, Adobe Systems Inc, San Jose, CA, USA) was used to automatically superimpose the images, using the “Scripts-Load Files into Stack” tool. The figure on top of the stack was placed in “multiply” view mode, and opacity was adjusted to permit proper visualization of the 2 images for the purpose of measurements. The effects of the different instruments on the canal walls were measured according to modifications obtained from previous study (15,16). Two evaluators working together and blinded to the groups performed all measurements. The amount of resin removed, i.e., the difference between the canal configuration before and after preparation, was determined for both the mesial and distal sides of the canal, in 1-mm increments, under high magnification and by using the Photoshop software ruler tool. The values obtained were corrected on the basis of the 1-mm scale generated by the stereomicroscope image capture system. The first measuring point was at the WL, i.e., the apical terminus of the canal (0 mm), and the last measuring point was 7 mm short of the WL (Fig. 2). This resulted in 8 measuring points for both the mesial and distal sides of the canal, a total of 16 measuring points per acrylic block (15). All measurements were made at right angles to the canal surface. If the difference between the mesial and distal measures at a given point was equal to 0, the canal was considered uniformly enlarged with no deviation, at least in the plane (mesiodistal) analyzed.

-Statistical analysis 

Data were analyzed by using the program BioEstat (MCT-CNPq, Belém, PA, Brazil), version 5.0. For comparison of the inter-group deviation, the Student’s-t test was used. The difference between the material removed from the mesial and distal walls of the canal at the 8 measuring points was compared by using repeated-measures analysis of variance (ANOVA). This initial analysis allowed identification of points where significant deviation occurred. For multiple comparisons, the Bonferroni test was used. Data were tested for normality before applying the parametric tests. The 5% level of statistical significance (*P*< 0.05) was established for all analyses.

## Results

The intragroup analysis revealed that both endodontic systems promoted some canal deviation at virtually all levels, as shown in  Table 1. This was verified by comparison of the amount of material that was removed from the mesial and distal walls of the artificial canals at the eight measuring points. Both systems removed similar amounts of material from the walls at almost all the measuring points, except at levels 0 and 4 that showed more deviation in TFA group (*P*<0.05), and at level 6 that showed more deviation in BT-RaCe group (*P*<0.05).

**Table 1:**
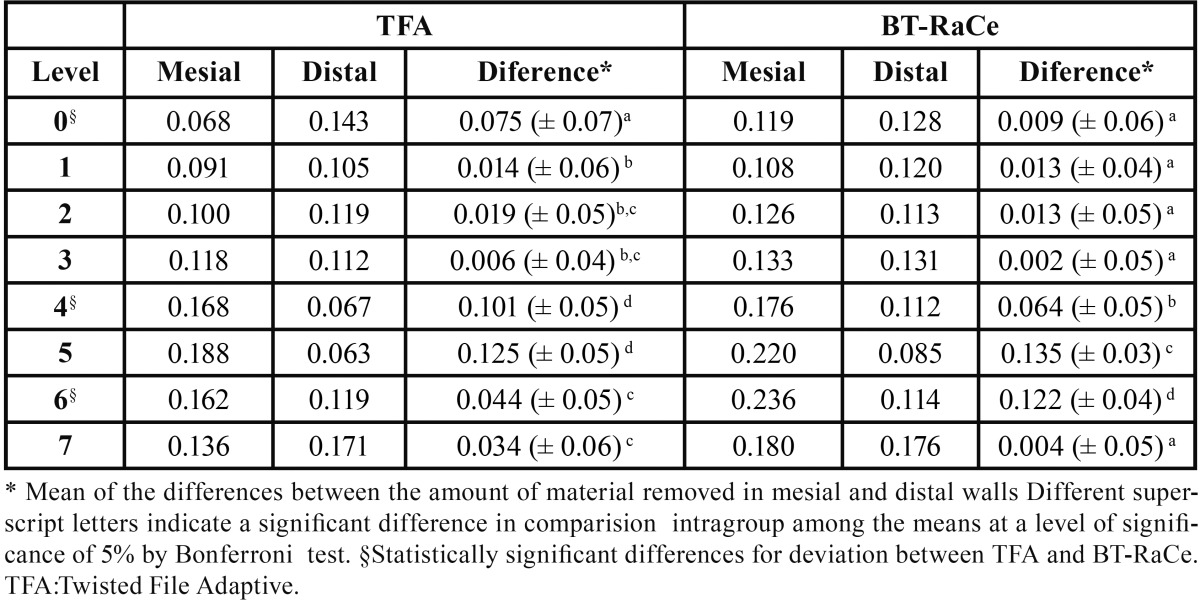
Mean amount of material removed (mm) at different levels from the apical terminus of simulated curved canals after preparation with two different nickel-titanium instruments.

## Discussion

In the current study, simulated curved root canals in resin blocks were used to standardize the experimental conditions of the study and to allow for direct visual comparison of the shaping ability of the endodontic instruments tested. These blocks have been widely used in recent and previous studies (15,17-19). Nonetheless, there are some limitations of this experimental model, such as the microhardness difference of the resin and dentin, and effect of heat generation promoted by instrumentation in the resin blocks, that favors the cutting this material (20-23). Due to the differences between resin and dentin, care should be taken when extrapolating the results (20-22).

At present, several studies have applied micro computed tomography as a method to evaluate the effects of canal preparation as it provides a three-dimensional view of instrumentation (23-26). One of the main difficulties with selecting a sample for the experiment is, however, to standardize teeth for inclusion in the research. By using resin blocks, the technique of superimposing preoperative and postoperative canal images is easily applied.

Both endodontic NiTi systems used in this study were developed recently, therefore, the performance of the TFA and BT-RaCe systems during preparation of simulated curved canals has not yet been compared. According to manufacturers, the instruments were designed to: enhance their properties; improve resistance to fracture; flexibility; and enable canal preparation with less deviation. The TFA system uses the Twisted File instrument that has a triangular cross-section, with the specific Elements Motor, featuring adaptive motion, changing the file motion based on the load applied. That is, it adapts to canal condition based on the amount of pressure on the instrument. When no load is applied it rotates continuously in a clockwise direction. When some load is applied it changes to a reciprocating motion, 370º forward and 50º backwards (12). The manufacturer of the newly introduced BT-RaCe system claims that it has: improved cutting efficiency; presents double cutting edges at the tip, provides safe instrumentation that ensures that the canal anatomy is respected with less apical transportation; and also provides electropolishing surface treatment. In this study, at the 0-mm level (the WL), BT-RaCe performed significantly better than TFA, confirming the BT-RaCe manufacturer’s information. In another study, Capar *et al.* (6), found similar results of the systems tested: Protaper Universal; Protaper Next TFA; Reciproc and WaveOne, after analyzing the curved mesial root preparations in mandibular molars, with no statistically significant difference among the groups. However, the cutting ability of endodontic files is a complex interrelationship of different parameters related to each instrument, such as the cross-sectional design; helical and rake angle; surface treatment of the instruments; and its kinematics with factors related to the mechanical behavior of the employed alloy, such as rigidity, flexibility, tenacity and hardness (6,12,27).

Although BT-RaCe showed better performance at 0 and 4-mm levels and the TFA system was better at 6-mm level, these findings can be probably explained due to the booster tip that the manufacturer claim about the BT-Race instrument. However, both systems presented deviation from the original canal anatomy at all analyzed levels, but the slightest deviation in points 6 promoted by TFA suggests greater security of this system to be used in the risk zone of mandibular molars (28). None of the NiTi tested systems was able to prepare the canals without transportation. Thus, although a significant improvement in canal preparation occurred with the advent of NiTi files when compared with stainless-steel instruments, it is still necessary to improve the performance of endodontic files during the instrumentation of curved root canals (20,21,29).

In the present study, the apical preparation was performed up to size 35/.04 at the WL because studies have shown that disinfection of the root canal can be improved with larger apical preparations (29-32) and, with the advent of NiTi instruments, this enlargement could be performed with reduced risks of procedural errors (33).

## Conclusions

In conclusion, the results of this study demonstrated that none of the NiTi tested systems was able to prepare curved canals without some deviation, as observed in previous studies (18,19,34). Therefore, there is still need for improvement in the instruments manufacturing aiming the better performance of endodontic files in curved root canals.
